# Supplementary material for: Mechanisms and attitudes in responsive healthcare for forced migrant communities: a qualitative study of transnational practice
Source: BMJ Open. 2025 Feb 17;15(2):e090211. doi: 10.1136/bmjopen-2024-090211 (PMC11836848; doi:10.1136/bmjopen-2024-090211)
Supplement: online supplemental file 1 [file bmjopen-15-2-s001.docx]

## Interview and discussion guides

### Discussion points for focus group sessions/interviews with people using services

**Reminder –** we are not from the Home Office; everything shared will remain confidential; conversation can stop or people can leave at any point.

1. **Are you happy to share with us how long you have been in the UK?**

*Helpful to know immigration status, level of English language, whether people have travelled alone or with family* ***but only*** *if volunteered*

1. **How important is your health to you? And how important is it to you that you are able to access health care when you need it?**

*Is it a priority or are other issues (possibly housing, asylum claims, work, family) more important?*

1. **Please tell us about a time when you have experienced good healthcare (this could be from when you were in your home country)**

*What does this look like? What can we learn from this? What matters to people?*

1. **How does this organisation show you that it cares?**

*Do you feel listened to and understood? Do you feel cared for?*

*What are your experiences of communicating with people in this service, including written communication and speaking with different people within the service?*

*What if someone doesn’t read or write in their language and are reliant on spoken communication? Or someone has no experience using or has no access to technology? Acknowledge that there may be a difference between getting what you want (for example antibiotics) and receiving care?*

*Are interactions with staff long enough? Is there enough time, particularly given the need for translation, for you to raise everything that you would like to discuss, to explain the issue, or to understand the advice or treatment plan being suggested?*

*Do staff show some understanding of your culture and the different life experiences you might have had as a refugee or asylum seeker?*

*Do you feel safe and believed and able to trust, including interpreters?*

*What difference has is made to you and your ability to receive health care when you need it? How independent do you feel in using health services, in making decisions or in speaking up, and asking for what you want or need to know? Has this been supported in some way by this service?*

1. **What would you describe as the most important aspects of the support that you receive from this service?**
2. **Beyond the service: Outside of this service, are there other things that have an impact on your health or experience of health services?**

*This could be positive or supportive (such as other services, friends or peers, community group or volunteers) or negative such as problems with housing, isolation, the asylum process, attitudes in the local area?*

1. ***Is there anything else that you would like to share or think we should know or be thinking about in relation to health care for refugees and people seeking asylum?***

### Interview guide – service leads

***Background and local context***

1. **Please could you provide an overview of [the service] and what you offer and tell us a little about how it has evolved?**

- *How long has the service been operating?*
- *What is the ‘patient’/ ‘client’ journey through and beyond the service?*
- *How do people know that this provision is here? How do they know how to find it, enter it, or how it works?*
- *Where is it located? Is there anything about these spaces, locations that present challenges or that are particularly appealing or helpful in reaching individuals?*
- *If R&AS are not the primary patient/care group how do they sit within services’ priorities?*
- *How does this service link with other activities/services that your organisation provides?*
- *What has been necessary to get it going, to keep it going. People, resources, relationships?*

1. **What sort of numbers of individuals are you working with at any given time? Does this reflect the number of refugees and asylum seekers in the area or are there individuals that you don’t reach or are supported elsewhere?**

- *Are there local conditions (local, national, geographical, political, economic, cultural etc.) that influence need or your/local response?*

1. **Who is involved in delivering what?**

- *How do you support your team to work with refugees and asylum seekers / do staff receive any training or supervision? How should we prepare a workforce to be responsive to a refugee/asylum seeking population? Are there sometimes challenges or anxieties when working cross-culture or -language?*
- *Are there specific skills, characteristics, qualities that you seek in your staff? Or a particular philosophy?*
- *Would it be fair to assume that there is a degree of experiential learning? Are you able to adapt / is this able to inform delivery?*

1. **Partnerships - what other agencies or services do you link with or work alongside and how crucial are they to what you deliver?**

- *How have these relationships evolved and how are they sustained?*
- *How do you manage and sustain the relationships and messaging with other services you might interact with in terms of prioritising care for refugees and asylum seekers and ensuring that their needs are being considered?*
- *What about how valued these are, levels of trust, communication, bureaucracy, flexibility?*
- *Are statutory services (or vice versa) prepared to work with you or are there barriers to sharing information, prioritising this population?*
- *Do you find you have to be ‘educators’ in terms of people’s rights or perform as specialists in this ‘field’?*
- *Are you able to influence systemic changes or are you ‘fire fighting’?*

1. **Funding and commissioning**

- *How are services resourced? Have you been able to access any statutory funding? E.g. money set aside for health care of resettlement refugees, or for a GP practice? How best to use that resource?*
- *Are there particular ways in which you have managed to influence commissioners* and what is it that commissioners ask of you*? Role of patient experience in that? Campaigning, local agendas, national agendas?*
- *Is there confidence that the service is sustainable?*
- *Are resources typically ascribed to each individual?*

**Responsive care for refugees and asylum seekers**

1. **How do you see the work that you and your colleagues do improving access to care for the people that you work with?**

- *What does this look like across the full journey of the ‘patient/’client’. From initial provision of information about the service, whether written or verbal, to physical access, patient/client interactions, provision of treatment or advice, referrals (where appropriate) and management of care/relationship.*
- *Have active steps been taken to support staff knowledge or skills in relation to: language, rights to healthcare, working cross-culturally (for example gender norms or stigma)? Consideration given to matching gender, ethnic background, language of workers and patients/clients?*
- *How is language and communication supported and managed throughout engagement with your service and potentially partners? What about non-readers? How are language support needs identified?*
- *Does advocacy play a role in your offer?*
- *What level of holistic care is there?*
- *Ho do you support people to understand how to use your service?*
- *Do you feel able to support people to have some agency around their health, decisions they might make or care they might seek?*

1. **If relevant, do you feel your practices should be absorbed by the NHS or do you see a benefit of it being provided by yourselves?**

*How much contact do you have with statutory health services?*

1. **What would you describe as the key critical elements of what you do or your approach?**

- *What matters most? Philosophy, principles, agenda, priorities, goals? Individually and/or as an organisation? What is it about your service that is most important central to what you do?*
- *What has been necessary to make these actions possible? Both the human and organisational processes.*
- *What have the challenges been? How they have been overcome? Who and what has been essential to this process?*
- *What outcomes matter most to the service? Maybe they are principles? Attitudes? Targets or objectives?*

1. **Do you feel able to be responsive to individuals needs or are you still constrained by other factors?**

- *How flexible are you able to be in terms of picking up on someone’s needs? For example, it may take time for confidence and trust in discussing certain issues. Or it might take time for an issue to manifest and it may do so in different ways, for example, trauma?*
- *Are there boundaries that you need to set with individuals and if so how do you maintain them – in terms of what you can do, are prepared to do, supporting people to adjust to a different health care system? How accommodating can you be of certain behaviours or ways of being.*

**Patient experience**

1. **What do you think matters most to the people that you work with?**

- *What are their priorities when they come into contact with your service? Do you think there are things they would like to see happen differently and if so, is that feasible?*
- *What do clients/patients say about their experiences with your service? How do you know that what you are doing is effective?*
- *Is it working for everyone you work with or just some groups? For example, less common languages/limited interpreters?*
- *How do you moderate fears and issues of trust?*
- *Have patients/clients played a part in shaping the service?*
- *Do you think association with the voluntary sector (or public sector) helps people to engage?*

1. **What about the broader aspects affecting the people who you work with?**

- *How do you manage people’s expectations and understandable distress, anxiety, anger?*
- *How do you support your staff with this?*

1. **Looking forward, what’s still to learn? What further developments of care do you consider important?**

### Interview guide – staff/volunteers

1. **How does your role fit into the broader service?**

*Could you describe the activities that you might support?*

1. **Have you had specific training or do you receive specific support to work in this role?**

*Are there sometimes challenges or anxieties when working cross-culture or -language?*

*How should we prepare a workforce to be responsive to a refugee/asylum seeking population?*

*Is there scope for experiential learning (confidence, skills, ways of working to develop over time)? Presumably that takes place on an individual level but what about collectively. Does this inform delivery?*

1. **Do you feel you bring specific skills or attributes that are important to the work that you do?**

- *Do you recognise these in your colleagues?*

1. **Partnerships - What other agencies or services do you link with or work alongside and how crucial are they to what you do?**

- *How are these relationships managed/sustained/valued/levels of trust and communication/bureaucracy/flexibility? Are statutory services (or vice versa) prepared to work with you or are there barriers to sharing information, prioritising this population?*
- *What contact do you have with the NHS (including GPs, hospital and community health services, pharmacy, ambulance service) or other refugee support services.*

**Responsive care for refugees and asylum seekers**

1. **How do you see the work that you and your colleagues do improving access to care for the people that you work with?**

- *What difference are you able to make to someone’s situation and experience of health care?*
- *What does this look like across the full journey of the ‘patient/’client’. From initial provision of information about the service, whether written or verbal, to physical access, patient/client interactions, provision of treatment or advice, referrals (where appropriate) and management of care/relationship.*
- *Have active steps been taken to support staff knowledge and skills in relation to: language, rights to healthcare, working cross-culturally (for example gender norms or stigma)? Consideration given to matching gender, ethnic background, language of workers and patients/clients? And does this effect the sorts of decisions that you make in your practice?*
- *How is language and communication supported and managed throughout engagement with your service and potentially partners? What about non-readers? How are language support needs identified?*
- *Does advocacy play a role in your offer?*
- *How far are you able to provide a holistic approach?*
- *How do you ensure people understand how to use your service?*
- *Do you feel able to support people to have some agency around their health, decisions they might make or care they might seek? Or is there an ongoing reliance on your involvement?*

1. **What would you describe as the most important elements of the work you do?**

- *What matters most? Philosophy, principles, agenda, priorities, goals? Individually and/or as an organisation? The most important factors or components that makes your service possible – what would they be?*
- *The key learning or message that you would share from the programme?*
- *What have the challenges been? How they have been overcome? Who and what has been essential to this process?*

1. **Do you feel able to be responsive to individuals needs or are you still constrained by other factors?**

- *Are there boundaries that you need to set with individuals and if so how do you maintain them – in terms of what you can do, are prepared to do, supporting people to adjust to a different health care system? For example, how accommodating can you be of certain behaviours or ways of being. Can it be sustained?*
- *How flexible are you able to be in terms of picking up on someone’s needs? For example, it may take time for confidence and trust in discussing certain issues. Or it might take time for an issue to manifest and it may do so in different ways, for example, trauma?*

**Patient experience**

1. **What do you think matters most to the people that you work with?**

- *What are your clients/patients priorities when they come into contact with your service? For example, is health care a priority? And what do people want from you?*
- *Do you think there are things they would like to see happen differently and if so, is that feasible?*
- *What do clients/patients say about their experiences with your service? How do you know that what you are doing is effective?*
- *Is it working for everyone you work with or just some groups? For example, less common languages/limited interpreters?*
- *Have patients/clients played a part in shaping the service?*
- *Do you think association with the voluntary sector (or public sector) helps people to engage?*
- *How do you ease people’s fears or issues of trust in the health care system?*

1. **What about the broader aspects affecting the people who you work with?**

- *For example, the asylum system. How do you manage people’s expectations and understandable distress, anxiety, anger?*

1. **Looking forward, are there further developments of care that you consider important?**
