# Supplementary material for: Mechanisms and attitudes in responsive healthcare for forced migrant communities: a qualitative study of transnational practice
Source: BMJ Open. 2025 Feb 17;15(2):e090211. doi: 10.1136/bmjopen-2024-090211 (PMC11836848; doi:10.1136/bmjopen-2024-090211)
Supplement: Supplementary file 3 [file bmjopen-2024-090211.reviewer_comments.pdf]

PEER REVIEW HISTORY

BMJ Open publishes all reviews undertaken for accepted manuscripts. Reviewers are asked to complete a checklist review form and are provided with free text boxes to elaborate on their assessment. These free text comments are reproduced below.

ARTICLE DETAILS

Title (Provisional)

Mechanisms and attitudes in responsive health care for forced migrant communities: a qualitative study of transnational practice

Authors

Robinson, Amy Rebecca; Khan, Ziaur Rahman A; Broadhurst, Kofi Amy; Nellums, Laura B; Renolds, Gisela; Payman, Bayan; Smith, Andrew

---

VERSION 1 - REVIEW

Reviewer 1

Name Vizheh, Maryam

Affiliation Australian Institute of Health Innovation (AIHI), Faculty of Medicine, Health and Human Sciences, Macquarie University

Date 24-Jul-2024

COI I declare no competing interests.

Thank you so much for giving me the opportunity to review this manuscript entitled: “Flexing care, a cup of tea and a Farsi welcome: mechanisms and attitudes in responsive health care for displaced communities. A qualitative study of transnational practice”. The manuscript aims to understand the opportunities and practices that can support responsive health care for forced migrant communities.

I find the topic important and the results interesting. However, there are fundamental flaws in writing. I have provided some suggestions to improve the clarity of work.

General comment:

The article needs an overall English proof. Especially using so many complex sentences made the paper difficult to follow.

Title:

I am not sure may I missed that. Why “Farsi welcome” in the title when there are participants (displaced participants) from other languages?

Abstract:

I suggest rewriting the abstract as the current version is really hard to follow.

In the sentence: “47 participants including people using or having used one of the five services”, please clearly indicate what you mean by “people”.

Introduction:

The introduction needs fundamental revisions. What do you mean by “responsive health”?

What do you mean by “displaced communities or Forced migrant community”? These concepts need definitions.

Methods

Please cite how many of the participants refused to be recorded and their reason if indicated.

What is the ID or number of the ethical approval?

The methods section is vague and too general. Please be more specific on several approaches used to collect data. How many people participated in the interview, or fo

Study design is not clear, and it is difficult to find different approaches to data collection and its related explanations. Please explain first the specific design of your study then explain its details. For example, for the first two paragraphs, what was the data collection approach? For the third paragraph explain clearly about the interviews and separately on service observations.

Please provide a brief explanation of the essentialist approach you took to analyze data.

Results

In the results section, it is important to mention from whom these quotations come. For instance, (refugee, 17 years old, man). The reader has no idea who on which cite (Australia, UK, or Belgium) has said them.

Discussion:

I cannot see the link between the title, introduction, methods, results, and discussion. From my point of view, each of the manuscript components somehow addresses different issues related to the topic.

References:

There are quite several old references. Please replace them with new ones.

Reviewer **2**

Name **Farrington, Rebecca**

Affiliation **University of Manchester Faculty of Biology, Medicine and Health, Division of Medical Education**

Date **01-Aug-2024**

COI **None.**

This is a very important paper - thank you for addressing the issues so comprehensively and from a holistic perspective. It has an accessible style and is very readable. It's refreshing to see positivity and solution-focussed work on this topic. Your reflections on non-attendance, trust and the slow burn of relationship-building resonated with my practice. In addition, describing advocacy in the form of multiplicity in professional practice was helpful. I appreciated the showcasing of examples of best practice eg the interpreter 'retranslating' back when culturally more appropriate words used.

Whilst I understand the challenge of a word count there were some areas I felt clarification on terminology could help. When using the umbrella term 'refugee' you generalise - the different levels of status are discussed in supplementary material, however acknowledgement that issues can be different for those without status would be good early on in the body of the text.

The accepted norm is to call oral translators 'interpreters' - this is clearer later in the paper but needs consistency. Was there any consideration that some participants may not be able to read in any language - forced migrants with low literacy are very marginalised - were they included? Also beware the assumptions of bilingually - it may be necessary for someone to declare or demonstrate competency in a language to safeguard complex communication.

The paper would be strengthened by thinking about the line between micro flexibility for good effect and 'tinkering' which may reinforce existing systemic barriers through accepting

substandard approaches. (See “That’s how we got around it” Tomkow et al). Slippage towards the use of non-state providers as an alternative rather than adjunct to care (possibly fuelled by strategic indifference) risks enacting the Inverse Care Law - you need clear responsibilities, boundaries, care pathways and accountability when working between organisations.

Connecting people from same communities can be amazing, but is not universally helpful because of political divides, social hierarchies, shame and trust - the paper would benefit acknowledging forced migrants need to have some autonomy over who exactly constitutes ‘their community’ and whether they wish them to be involved in their care - I’ve seen lots of assumptions made that have turned people away.

VERSION 1 - AUTHOR RESPONSE

Reviewer: 1

Dr. Maryam Vizheh, Australian Institute of Health Innovation (AIHI), Faculty of Medicine, Health and Human Sciences, Macquarie University

Comments to the Author:
Thank you so much for giving me the opportunity to review this manuscript entitled: “Flexing care, a cup of tea and a Farsi welcome: mechanisms and attitudes in responsive health care for displaced communities. A qualitative study of transnational practice”. The manuscript aims to understand the opportunities and practices that can support responsive health care for forced migrant communities.

I find the topic important and the results interesting. However, there are fundamental flaws in writing. I have provided some suggestions to improve the clarity of work.

General comment:

1. **The article needs an overall English proof. Especially using so many complex sentences made the paper difficult to follow.**

   Thank you for your comment, we have sought to address these concerns and have conducted a thorough ‘English proof’ for readability though do note that reviewer two has highlighted the accessible style and readability of the manuscript.

2. **Title:**
   I am not sure may I missed that. Why “Farsi welcome” in the title when there are participants (displaced participants) from other languages?

   This had been used as an example only of the sort of welcome services gave to their services users and should have been included in quotations. In the event we have amended the title to better reflect the format requested by the journal.

Abstract

3. **I suggest rewriting the abstract as the current version is really hard to follow.**

Thank you for your comments. We have made some edits to the abstract and hope that we have addressed this concern.

4. **In the sentence: "47 participants including people using or having used one of the five services", please clearly indicate what you mean by "people".**

   Thank you for highlighting this point. We have clarified this sentence with some edits (p.2).

Introduction

5. **The introduction needs fundamental revisions. What do you mean by "responsive health**"?

   We have now included a definition as to how we define responsive health care (p.4).

6. **What do you mean by "displaced communities or Forced migrant community"? These concepts need definitions.**

   Thank you for highlighting this. A definition has been added to p.3.

Methods

7. **Please cite how many of the participants refused to be recorded and their reason if indicated.**

   This related to two individuals and no explanation was provided. We have provided further detail on p.5.

8. **What is the ID or number of the ethical approval?**

   This has been added on p.6.

9. **The methods section is vague and too general. Please be more specific on several approaches used to collect data. How many people participated in the interview, or fo**

   The numbers of people interviewed are detailed in the results section (p.6).

10. **Study design is not clear, and it is difficult to find different approaches to data collection and its related explanations. Please explain first the specific design of your study then explain its details. For example, for the first two paragraphs, what was the data collection approach? For the third paragraph explain clearly about the interviews and separately on service observations.**

    Please provide a brief explanation of the essentialist approach you took to analyze data.

    Some edits have been made to this section which we hope makes our approach to analysing the data clearer (p.5).

Results

11. **In the results section, it is important to mention from whom these quotations come. For instance, (refugee, 17 years old, man). The reader has no idea who on which cite (Australia,**

UK, or Belgium) has said them.

Thank you for your comment. Though we have made some minor edits and do recognise the benefit of providing identifiers to quotations we have found this difficult to address. Confidentiality was incredibly important in this study and includes the risk of damaging relationships between services and statutory providers/contractors on which the services heavily rely. As we have named and described the services, associating quotes with a service or a role could enable identification of services as could the simple use of an anonymous identifier, such as a number which, if cross referenced across quotations would also risk identification of individuals/services. This is a practice discouraged by our patient involvement advisor and something that we feel would jeopardise our commitment to the services involved.

We have tried to make it very clear where quotations or ideas put forward in quotations were shared by others and where they were ideas expressed by a singular person or service. Reporting service user interviews was difficult because of anonymity and it was important that neither readers nor services (and partner services) could identify participants.

Discussion

12. **I cannot see the link between the title, introduction, methods, results, and discussion. From my point of view, each of the manuscript components somehow addresses different issues related to the topic.**

    We have conducted a full edit of the manuscript with this in mind which we hope has addressed these concerns.

    References:

13. **There are quite several old references. Please replace them with new ones.**

    Thank you for highlighting this, however, we are not clear on which references are of concern.

Reviewer: 2

Dr. Rebecca Farrington, University of Manchester Faculty of Biology, Medicine and Health
Comments to the Author:

This is a very important paper - thank you for addressing the issues so comprehensively and from a holistic perspective. It has an accessible style and is very readable. It's refreshing to see positivity and solution-focussed work on this topic. Your reflections on non-attendance, trust and the slow burn of relationship-building resonated with my practice. In addition, describing advocacy in the form of multiplicity in professional practice was helpful. I appreciated the showcasing of examples of best practice eg the interpreter 'retranslating' back when culturally more appropriate words used.

1. **Whilst I understand the challenge of a word count there were some areas I felt clarification on terminology could help. When using the umbrella term 'refugee' you generalise - the different levels of status are discussed in supplementary material, however acknowledgement that issues can be different for those without status would be good early on in the body of the text.**

Thank you for this feedback which we appreciate and have taken on board. We have now included a definition (p.3) which also acknowledges the challenges and implications of using classifications. We have also replaced the term ‘refugee’ with ‘forced migrant’ to better encompass the heterogeneity in status ascribed to people fleeing conflict and persecution.

2. **The accepted norm is to call oral translators ‘interpreters’ - this is clearer later in the paper but needs consistency. Was there any consideration that some participants may not be able to read in any language - forced migrants with low literacy are very marginalised - were they included? Also beware the assumptions of bilingually - it may be necessary for someone to declare or demonstrate competency in a language to safeguard complex communication.**

   Thank you for highlighting the inconsistency. This has been addressed (p.4, 11, 12, 18, 19) except where it was the term used by a participant.

   We did consider that some participants may not have literacy in their native language, did encourage inclusion via service links and would have worked with an interpreter to support the consenting process if this had been the case. In the event, all participants did have native language literacy.

   Consideration and practices to support health care in the context of low literacy was picked up quite a bit in our associated review which will be published shortly.

3. **The paper would be strengthened by thinking about the line between micro flexibility for good effect and ‘tinkering’ which may reinforce existing systemic barriers through accepting substandard approaches. (See “That’s how we got around it” Tomkow et al). Slippage towards the use of non-state providers as an alternative rather than adjunct to care (possibly fuelled by strategic indifference) risks enacting the Inverse Care Law - you need clear responsibilities, boundaries, care pathways and accountability when working between organisations.**

   Thank you very much for drawing our attention to the concept of tinkering and the work of Tomkow et al. This is an important distinction which we have worked into the discussion (p.19).

4. **Connecting people from same communities can be amazing, but is not universally helpful because of political divides, social hierarchies, shame and trust - the paper would benefit acknowledging forced migrants need to have some autonomy over who exactly constitutes ‘their community’ and whether they wish them to be involved in their care - I’ve seen lots of assumptions made that have turned people away.**

   Thank you for this comment and for encouraging wider thought on this. We have made some additions which highlight the need for nuance in this thinking (p18).

VERSION 2 - REVIEW

Reviewer 1

Name Vizheh, Maryam

Affiliation Australian Institute of Health Innovation (AIHI), Faculty of Medicine, Health and Human Sciences, Macquarie University

Date 09-Dec-2024

COI

I believe authors did good work to improve the quality and especially clarity of their manuscript.

General comment

This is just a comment for the future works of authors. It is really good to provide explanations which you have brought in the main text to the response to reviewers' document, too. Your answer to the comment with page and line number is required. Also provide the response in here. For example, if you provide any explanations about ... bring that in response to the reviewers. So, the document is standalone informative. But in the current version you just say, we addressed that in page ... which is not informative.

English: again, the language is very difficult to understand, and I cannot see any significant difference compared to the previous version. Please try to use less complex sentences.

Introduction: Bring the definition of "responsive healthcare" into the early sections of introduction.

-I do not think bringing the definition of forced population in the framework is appropriate. Just bring it in the introduction.

Methods:

Page 6: in sentence "included people with "experiences" of forced migration", experience dictation is incorrect.

References: I am surprised of using so many old references. More than 34 references are too old while authors stated "we are not sure which references you think are old"! the manuscript has some references ranged from 1988!Those in which theories are used like those of Braun or Mercer are acceptable. But a systematic review from 2005??!!

We often prefer up-to-date references in the last 5 years except in some special cricumstances.

Please replace them. I provided some examples:

1. Clark RC, Mytton J. Estimating infectious disease in UK asylum seekers and refugees: a systematic review of prevalence studies. *J Public Health* 2007;29:420–8.
2. Drury J, Williams, R. Children and young people who are refugees, internally displaced persons or survivors or perpetrators of war, mass violence and terrorism. *Curr Opin Psychiatry*. 2012;25(4):277-284. doi:10.1097/YCO.0b013e328353eea6
3. Fazel M, Wheeler J, Danesh J. Prevalence of serious mental disorder in 7,000 refugees resettled in Western countries: A systematic review. *The Lancet*. 2005;365:1309–1314.
4. Aspinall P. *Hidden needs. Identifying key vulnerable groups in data collections: vulnerable Migrants, Gypsies and Travellers, Homeless People, and sex workers.* 2014. https://assets.publishing.service.gov.uk/government/uploads/system/uploads/attachment_data/file/287805/vulnerable_groups_data_collections.pdf
5. Cheng IH, Drillich A, Schattner P. Refugee experiences of general practice in countries of resettlement: a literature review. *Br J Gen Pract*. 2015;doi:doi.org/10.3399/bjgp15X683977.

6. Mann JM, Gostin L, Gruskin S, Brennan T, Lazzarini Z, Fineberg HV. Health and Human Rights. *Health and human Rights*. 1994;1:6-23.

7. Lustig SL, Kia-Keating, M., Knight, W.G., Geltman, P., Ellis, H., Kinzie, J.D., Keane, T., Saxe, G.N. Review of child and adolescent refugee mental health. *Journal of the American Academy of Child and Adolescent Psychiatry*. 2004;43:24–36.

8. Morris MD, Popper, S.T., Rodwell, T.C., Brodine, S.K., Brouwer, K.C. Healthcare barriers of refugees post-resettlement. *J Community Health*. 2009;34(6)doi:10.1007/s10900-009-9175-3

9. Bauer AM, Alegría, M. Impact of patient language proficiency and interpreter service use on the quality of psychiatric care: a systematic review. *Psychiatr Serv*. 2010;61(8):765–73. doi:10.1176/ps.2010.61.8.765)

10. Gadeberg AK, Norredam, M. Urgent need for validated trauma and mental health screening tools for refugee children and youth. *Eur Child Adolesc Psychiatry*. 2016;25:929–931. doi:10.1007/s00787-016-0837-2

11. Burnett A, Peel, M. Health needs of asylum seekers and refugees. *BMJ*. 2001;322(7285):544–547.

12. 19. Papadopoulos I, Lees S, Lay M, Gebrehiwot A. Ethiopian refugees in the UK: migration, adaptation, and settlement experiences and their relevance to health. *Ethn Health*. 2004;9(1):55–73.

13. Daniel M , Devine C , Gillespie R ea. *Helping new refugees integrate into the UK: baseline data analysis from the survey of New Refugees.* . 2010. https://www.gov.uk/government/uploads/system/uploads/attachment_data/file/116069/horr36-report.pdf

14. Harris MF, Telfer BL. The health needs of asylum seekers living in the community. *Med J Aust*. 2007;175(11–12):589–592.

15. Montgomery E, Foldspang, A. Discrimination, mental problems and social adaptation in young refugees. *European Journal of Public Health*. 2008;Volume 18(2):156–161. doi:10.1093/eurpub/ckm073

16. Erasmus E. The use of street-level bureaucracy theory in health policy analysis in low- and middle-income countries: a meta-ethnographic synthesis. *Health Policy and Planning*. 2014;29(suppl_3):iii70-iii78. doi:10.1093/heapol/czu112

17. Eisenbruch M. The mental health of refugee children and their cultural development. *International Migration Review*. 1988;22:282-300.

18. Davies AA, Blake C, Dhavan P. Social determinants and risk factors for non-communicable diseases (NCDs) in South Asian migrant populations in Europe. *Asia Europe J*. 2011;8(4):461–73.

19. Bemak F, Chung, R.C. Immigrants and refugees. In: Leong FTL, Nagayama Hall, G.C., McLoyd, V.C., et al., ed. *APA Handbook of Multicultural Psychology, Vol 1: Theory and Research*. American Psychological Association; 2014:503–517.

20. Vandemark LM. Promoting the sense of self, place, and belonging in displaced persons: the example of homelessness. . *Arch Psychiatr Nurs* 2007;21(5):241-8. doi:10.1016/j.apnu.2007.06.003. PMID: 17904481.

21. Schonfeld DJ, Demaria, T. Providing Psychosocial Support to Children and Families in the Aftermath of Disasters and Crises. *Pediatrics*. 2015;136(4):e1120–e1130. doi:10.1542/peds.2015-2861

22. UN. United Nations Resolution 70/1. Transforming our world: the 2030 agenda for sustainable development. New York: United Nations; 2015.

23. Fiddian-Qasmiyeh E, Loescher G, Long K, Sigona NE. *The Oxford Handbook of Refugee and Forced Migration Studies.* Oxford University Press; 2014.

24. Porter M, Haslam N. Predisplacement and postdisplacement factors associated with mental health of refugees and internally displaced persons. A meta-analysis. *J Am Med Assoc*. 2005;294:602–612.

25. Bronfenbrenner U. Toward an experimental ecology of human development. *Am Psychol*. 1977;32:513–531.
26. Weiss L, Gany F, Rosenfeld P, et al. Access to multilingual medication instructions at New York City pharmacies. *J Urban Health*. Nov 2007;84(6):742-54. doi:10.1007/s11524-007-9221-3
27. Service RW. Book Review: Corbin, J & Strauss, A (2008). Basics of Qualitative Research: Techniques and Procedures for Developing Grounded Theory (3rd ed.). Thousand Oaks, CA: Sage. *Organizational Research Methods*. 2009;12(3):614-617. doi:10.1177/1094428108324514
28. Russell J, Greenhalgh T, Boynton P, M R. Soft networks for bridging the gap between research and practice: illuminative evaluation of CHAIN. *BMJ*. 2004;15(328)doi:10.1136/bmj.328.7449.1174
29. Polanyi M. *The tacit dimension*. Anchor Day; 1962.
30. Bischoff A, Bovier PA, Rrustemi I, Gariazzo F, Eytan A, Loutan L. Language barriers between nurses and asylum seekers: their impact on symptom reporting and referral. *Soc Sci Med*. Aug 2003;57(3):503-12. doi:10.1016/s0277-9536(02)00376-3
31. Flores G, Laws MB, Mayo SJ, et al. Errors in Medical Interpretation and Their Potential Clinical Consequences in Pediatric Encounters. *Pediatrics*. 2003;111(1):6-14. doi:10.1542/peds.111.1.6
32. Fichter JH. *Sociology*. Chicago University Press; 1957.
33. EuropeanCommission. *The Common Basic Principles for Immigrant Integration Policy in the*
34. *EU*. 2004. Accessed 2 February 2023. https://ec.europa.eu/migrant-integration/library-document/common-basic-principles-immigrant-integration-policy-eu_en

---

Reviewer **2**

Name **Farrington, Rebecca**

Affiliation **University of Manchester Faculty of Biology, Medicine and Health, Division of Medical Education**

Date **07-Dec-2024**

COI

Thank you for taking on board the comments in the initial review. This is an important piece of work highlighting the complexity of working with forced migrants and acknowledging the wide range of possibilities for respectful and culturally humble approaches to care.

VERSION 2 - AUTHOR RESPONSE

Thank you very much for the additional comments provided by Reviewer 1. We have addressed these against each point below and made edits as described in the manuscript.

General comment
(1) English: again, the language is very difficult to understand, and I cannot see any significant difference compared to the previous version. Please try to use less complex sentences.

We have conducted a further readability proof of the manuscript with attention to the more complex

sentences as suggested. Edits fall predominantly in the Introduction and Discussion. We hope that we have now adequately addressed these concerns.

(2) Introduction: Bring the definition of “responsive healthcare” into the early sections of introduction.

We have now included reference to responsive health care in the second paragraph of the introduction (p.3).

(3) I do not think bringing the definition of forced population in the framework is appropriate. Just bring it in the introduction.

We have moved this definition to the concluding paragraph of the introduction (p.3).

Methods
(4) Page 6: in sentence “included people with “experiences” of forced migration”, experience dictation is incorrect.

Thank you for highlighting this error which has been corrected.

(5) References: I am surprised of using so many old references. More than 34 references are too old while authors stated “we are not sure which references you think are old”! the manuscript has some references ranged from 1988!Those in which theories are used like those of Braun or Mercer are acceptable. But a systematic review from 2005??!!
We often prefer up-to-date references in the last 5 years except in some special cricumstances. Please replace them. I provided some examples:

Thank you for identifying the references with which you have concerns. Though barriers to accessing care for forced migrants are well documented these are generally broad studies focusing largely on access to primary and mental health care. Beyond this, and in relation to more specific health needs and conditions and cultural intersections, forced migrants remain underrepresented in the literature. We have made some changes in respect of some of the references highlighted (please see below) however to our knowledge the studies referenced are a good representation of the existing literature.

• We have brought in some additional studies, Samari (2024) and Nematy (2023) to support earlier work from Clark (2007) and Drury (2012).
• Blackmore (2020), supports Fazel (2005).
• We have removed Eisenbruch (1988). This was already supported by Nellums (2018).
• We have referenced, Krystallidou 2024 rapid review looking at communication in refugee and migrant mental healthcare which touches on some of the evidence we have referenced by Bischoff 2003 and Flores 2003.
• The majority of the earlier texts (Bronfenbeener 1977; Polyanyi 1962; Fichter 1957; Fiddian-Qasmiyeh 2014) are important thematic and theoretical texts that we believe support our arguments.
